# Chemical screening identifies the anticancer properties of *Polyporous tuberaster*

**DOI:** 10.7150/jca.86304

**Published:** 2023-07-09

**Authors:** Eun Seon Song, Moon Kyoung So, Hyon Jin Park, Haneur Lee, Yun Haeng Lee, Myeong Uk Kuk, Jiho Park, Hyung Wook Kwon, Jaehyuk Choi, Joon Tae Park

**Affiliations:** 1 Division of Life Sciences, College of Life Sciences and Bioengineering, Incheon National University, Incheon, Korea.; 2 Convergence Research Center for Insect Vectors, Incheon National University, Incheon 22012, Korea.

**Keywords:** *polyporus tuberaster*, mycelium culture extract, chemical screening

## Abstract

Most conventional anticancer drugs cause resistance to chemotherapy, which has emerged as one of the major obstacles to cancer treatment. In order to address this issue, efforts have been made to select new anticancer compounds from natural sources. The aim of this study is to identify novel anticancer compounds from mycelial culture extracts belonging to *Polyporus tuberaster* (*P. tuberaster*). Here, we found that mycelial culture extracts of *P. tuberaster* cultured in PDB medium (pt-PDB) effectively inhibited cancer cell growth. pt-PDB reduced the growth of cancer cells through apoptosis induction and S-phase arrest. The anticancer efficacy of pt-PDB was not to limited to one type of cancer. Furthermore, unlike traditional anticancer medications, pt-PDB did not increase the proportion of side population (SP) cells, which plays a key role in the development of chemoresistance. Taken together, we discovered a novel anticancer drug candidate that has anticancer properties without increasing the proportion of SP cells. This new drug candidate can be used for the treatment of cancer, especially chemoresistant malignancies, and will provide a breakthrough in the treatment of chemoresistant cancer.

## Introduction

Mushrooms have been historically valued as a food source due to their low fat and high protein contents, as well as a functional food beneficial to human health 1. Mushrooms produce primary metabolites and secondary metabolites 2. Primary metabolites include nucleic acids and proteins, while secondary metabolites include polysaccharides, terpenoids, alkaloids, lectins, and metal chelators 3. Secondary metabolites, in particular, have been found to possess potent biological activities, including antibacterial, antiviral, and anticancer properties 3. Among the activities, anticancer effect has received much attention, and several clinical trials are being conducted to evaluate the efficacy of the mushroom-derived compounds 4. For instance, Lingzhi mushroom (*Ganoderma lingzhi*) was demonstrated in a phase 2 clinical trial to help maintain the quality of life of cancer patients after chemotherapy 5. Similarly, orally administered *Ganoderma lucidum* has been shown to be beneficial in the treatment of colorectal cancer patients by acting as an immunomodulator 6. In another phase 1 clinical study, the button mushroom was found to reduce prostate-specific antigen levels in the patients with recurrent prostate cancer 7. Taken together, the promising results from these clinical studies suggest that mushrooms with anticancer properties may hold great potential for the treatment of cancer patients either as a standalone therapy or in combination with conventional anticancer drugs.

The life cycle of mushrooms consists of two distinct stages: the long-lived mycelial stage and the short-lived fruiting body stage 8. As fruiting bodies are cultured on a solid medium, large-scale cultivation requires large space and time. In contrast, mycelium is composed of tubular threads known as hyphae, and possesses a porous structure, rendering it suitable for liquid cultivation 9. Recently submerged culture of mycelia has been introduced for mass cultivation of mycelia, thereby enabling the low-cost production of secondary metabolites on a large scale 9. Maximizing the mass production of secondary metabolites through liquid culture of mycelia can be achieved by manipulating process factors and medium components 10. Therefore, in order to mass-produce secondary metabolites at low cost, liquid culture using mycelia is essential.

Drug resistance is a major impediment to successful chemotherapy and continues to pose a challenge in cancer treatment 11. Despite remarkable advances in conventional chemotherapy, the overall mortality rate of cancer patients remains high due to the emergence of drug-resistant cells 12. Numerous studies are ongoing to identify novel mechanisms addressing drug resistance. Recent studies have revealed that side population (SP) cells play an important role in developing drug resistance and inducing relapse of all malignancies during chemotherapy 13, 14. SP cells have multidrug resistance due to overexpression of the ATP-binding cassette (ABC) transporter, which plays a role in drug efflux 15, 16. Given these results, finding drugs that do not increase the proportion of SP cells may reduce drug resistance, making it an effective chemotherapy option for cancer patients.

The genus *Polyporus* belong to the family *Polyporaceae* is a kind of wood rot mushroom 17. Recently, *Polyporous parvovarius* was found to inhibit tumor cell proliferation and promote apoptosis 18. However, investigations into the anticancer properties of other *Polyporus* species have not been extensively conducted despite the genus consisting of over 250 species. This study aimed to determine whether mycelial culture extracts of *Polyporus tuberaster* (*P. tuberaster*) possess anticancer properties. Our findings demonstrate that *P. tuberaster* mycelium culture extracts exhibit anticancer properties without inducing drug resistance.

## Materials and Methods

### Mycelium liquid culture

*Polyporus tuberaster* (Accession number: KMRB 18083112) used in this study was sourced from Korea Mushroom Resource Bank (KMRB, Seoul, Korea). Four media were used for mycelium culture (Table 1).

Extracts were produced from mycelium cultures as described in previous studies 19. Briefly, precultured mycelial discs (5 mm) were inoculated into each Erlenmeyer flask with 700 ml of medium. The flasks were then incubated for 60 days at 25 °C while being shaken at 150 × *g*. To obtain a culture medium, a liquid culture medium containing mycelium was filtered through Miracloth (475855-1R; Calbiochem, La Jolla, CA, USA). The culture filtrate was separated and freeze-dried. Dried filtrates were soaked in 100% ethanol. After filtering the extract with 0.45 μm membrane filters (HAWP05000; Sigma) to remove the remaining residue, the ethanol was evaporated. The remaining extract was diluted in 500 mg/ml dimethyl sulfoxide (41639; Sigma).

### Cell Culture

Seven different cell lines were used and detailed information is provided in Table 2. Cells was cultured as described previously 18.

### Cell proliferation assay

At 2,000 cells per well, HeLa cells were plated on 96-well plates. Extracts of *P. tuberaster* mycelium grown in four separate culture media (DYB, MEB, MYB and PDB) were diluted to a final concentration of 0.1 mg/ml. Four days after starting drug treatment, cell proliferation was assessed. A cell proliferation assay based on a DNA content was utilized to measure the number of cells 27. Briefly, 50 μl of 0.2% SDS was added to each wells and incubated at 37 °C for 1 h. Then, 150 μl of SYBR Green I nucleic acid gel stain (1:1,000 in D.W., S-7567, Molecular Probes, Eugene, OR, USA) was added to each of the 96 wells. Fluorescence intensity was quantified to determine cell number using a VICTOR Multilabel Plate Reader (2030-0050; PerkinElmer, Waltham, MA, USA).

### Determining pt-PDB's cytotoxicity

At 2,000 cells per well, HeLa cells were plated on 96-well plates. At dilutions of 0.1 to 0.5 mg/ml, pt-PDB was used. A cell proliferation assay based on a DNA content was utilized to measure the number of cells 27. Four days after starting drug treatment, cell proliferation was assessed. Cell proliferation was measured at each dose and its values were normalized to those in DMSO in order to evaluate the viability of the cells.

### Determining pt-PDB's optimal concentration to inhibit cell proliferation

At 2,000 cells per well, HeLa cells were plated on 96-well plates. Cell proliferation was assessed 1, 2, 3 and 4 days after treatment at concentrations of 0.02 to 0.1 mg/ml.

### Trypan blue staining

Trypan blue staining with 0.4% trypan blue was assessed by a Cedex HiRes Analyzer (05650216001; Roche, Basel, Switzerland). Brightfield cell images were automatically captured on a Cedex HiRes analyzer.

### Apoptosis assay

Apoptosis assay using the FITC Annexin V Apoptosis Detection Kit (556547; BD Biosciences, Franklin Lakes, NJ, USA) was performed as previously described 28.

### Cell cycle assay

Cell cycle assay was performed as previously described 29. In brief, cells were stained with 50 g/ml propidium iodide (PI, P4170-10MG; Sigma) after being fixed with 70% ethanol.

### Western blot analysis

Western blot analysis was performed as described previously 18. Primary antibodies used in this study p21Cip1 antibody (sc-6246; 1:500 dilution; Santa Cruz Biotechnology, Dallas, TX, USA), phospho-retinoblastoma (Rb) antibody (sc-271930; 1:500 dilution; Santa Cruz Biotechnology), phospo-ERK antibody (9101s; 1:500 dilution; Cell signaling technology, Danvers, MA, USA), p-MEK antibody (9121; 1:500 dilution; Cell signaling technology), Cleaved-Caspase 8 (9746s; 1:500 dilution; Cell signaling technology), Cleaved-Caspase 9 (9502S; 1:500 dilution; Cell signaling technology) and HRP-conjugated β-actin (sc47778; 1:1000 dilution; Santa Cruz Biotechnology). Secondary antibodies used in this study HRP conjugated anti-mouse antibody (sc-516102; 1:1000 dilution; Santa Cruz Biotechnology) and HRP conjugated anti-rabbit antibody (sc-2357; 1:1000 dilution; Santa Cruz Biotechnology).

### Side population (SP) assay

5 μg/ml Hoechst 33342 (H3570; Thermo Scientific, Waltham, MA, USA) or 5 μM verapamil (V4629; Sigma) were applied to 1 × 10^6^ cells for 90 min at 37 °C. Cells were stained with 1 μg/ml PI on ice for 5 min after being washed with PBS. LSR II (BD Biosciences) was used to analyze flow cytometry data.

### Statistical analyses

The statistical analysis was carried out using a statistical software program (SigmaPlot 12.5; Systat Software, San Jose, CA, USA). Student's t-test was used to determine whether differences were significant.

## Results

### Chemical screening of *P. tuberaster* mycelium culture extracts for antitumor activity

**Figure 1:**
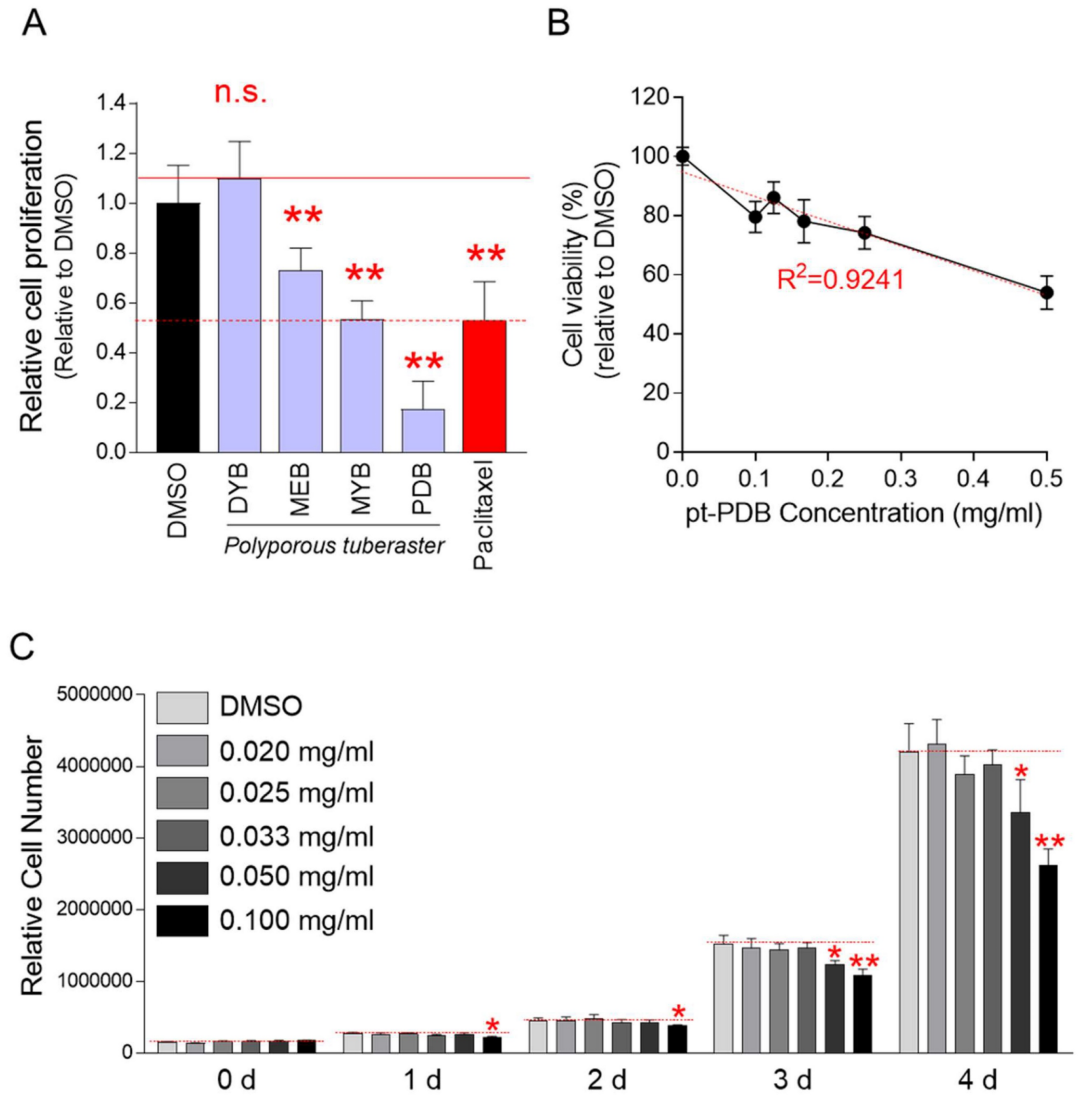
Chemical screening of *P. tuberaster* mycelium culture extracts for antitumor activity. (A) Four extracts were produced from *P. tuberaster* mycelium grown in four distinct culture media (DYB, MEB, MYB and PDB). Hela cells were treated with each extract at a dose of 0.1 mg/ml. The cell proliferation inhibitory effect was evaluated on the 4th day of treatment. Paclitaxel was used as a positive control group. Student t-test, **P < 0.01, student t-test. Means ± S.D., *n* = 10. (B) Determining pt-PDB's cytotoxicity. Cell viability was assessed by measuring cell proliferation at 0.1-0.5 mg/ml pt-PDB concentrations and comparing the results to those in DMSO. Means ± S.D., *n* = 10. R^2^ (coefficient of determination) = 0.9241. (C) Determining pt-PDB's optimal concentration to inhibit cell proliferation. Cell proliferation was assessed 1, 2, 3 and 4 days after treatment at concentrations of 0.02 to 0.1 mg/ml. *P < 0.05, **P < 0.01, student t-test. Means ± S.D., *n* = 10.

A screening technique, a method of counting cells based on their DNA content, was used to find mycelial culture extracts of *P. tuberaster* that significantly slowed the growth of Hela cells 30. Four extracts were produced from the mycelial culture of *P. tuberaster*, which was grown in four distinct media: DYB, MEB, MYB and PDB. Hela cells were treated with each extract at a dose of 0.1 mg/ml. The cell proliferation inhibitory effect was assessed on the 4th day of treatment. Paclitaxel, a drug commonly used to treat various types of tumors was used as a positive control 31. The cell proliferation inhibition effects of mycelial culture extracts from MEB, MYB, and PDB media were significant when compared to the DMSO control (Fig. 1A). However, the mycelial culture extract in the DYB did not significantly inhibit cell proliferation. Mycelial culture extract in PDB medium (a.k.a., pt-PDB) was selected for further investigation because it reduced cell proliferation even more effectively than paclitaxel (positive control) (Fig. 1A).

Although pt-PDB showed an inhibitory effect on cell growth, it was not rule out the possibility that this effect was due to cytotoxicity rather than an anticancer effect. To rule out this option, cell viability was assessed at doses of 0.1-0.5 mg/ml of pt-PDB. The decrease in cell viability was concentration dependent (Fig. 1B). R^2^ value, coefficient of determination, was 0.9241 indicating that the ability of pt-PDB to limit cell proliferation was not a result of cytotoxicity but rather an anticancer effect (Fig. 1B).

We then investigated the optimal pt-PDB concentration that could effectively reduce cell proliferation. Cell proliferation was assessed 0, 1, 2, 3 and 4 days after treatment at concentrations of 0.02 to 0.1 mg/ml. Compared to the DMSO control, pt-PDB concentrations below 0.05 mg/ml did not show a significant decrease in cell proliferation (Fig. 1C). However, a pt-PDB concentration of 0.05 mg/ml significantly decreased cell proliferation from day 3 after drug treatment (Fig. 1C). Moreover, the pt-PDB concentration of 0.1 mg/ml used for chemical screening showed a significant decrease in cell proliferation from day 1 after drug treatment (Fig. 1C). Since a pt-PDB concentration of 0.1 mg/ml inhibited cell proliferation from day 1 after drug treatment, 0.1 mg/ml was selected as the optimal pt-PDB concentration and applied to all subsequent experiments.

### pt-PDB exerts anticancer activity through apoptosis induction and S-phase arrest

**Figure 2:**
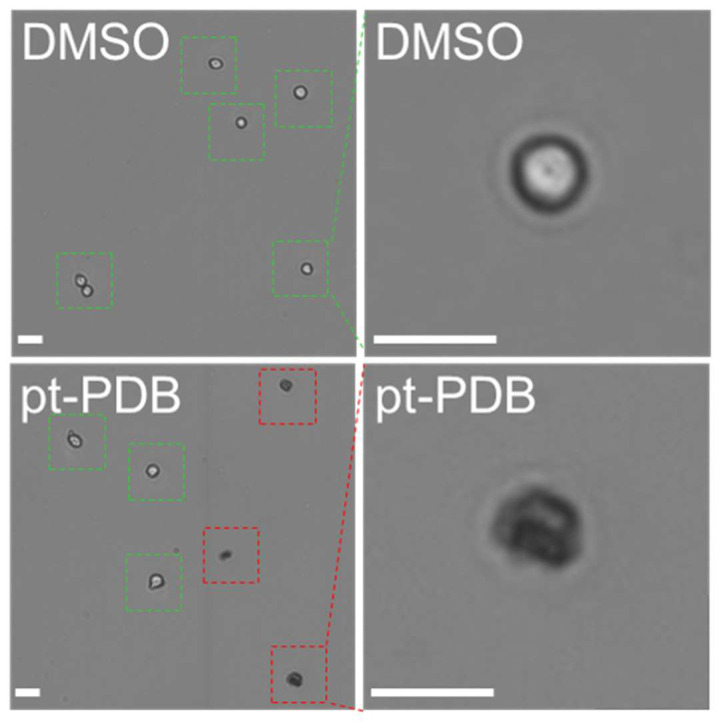
Determination of anticancer activity of pt-PDB by trypan blue staining. Cells were stained with 0.4% trypan blue and brightfield cell images were captured using a Cedex HiRes analyzer. Green squares represent live cells and red squares represent dead cells. Scale bar 10 μm.

The anticancer activity of pt-PDB was determined by observing whether pt-PDB effectively kills cancer cells. Trypan blue staining was used to discriminate dead cells from live cells after pt-PDB treatment 32. In the DMSO control, all cells were negative for trypan blue staining indicating live cells (Fig. 2; green squares represent live cells). However, in the pt-PDB group, cells positive for trypan blue staining were observed along with cells negative for trypan blue staining (Fig. 2; green squares represent live cells and red squares represent dead cells).

**Figure 3:**
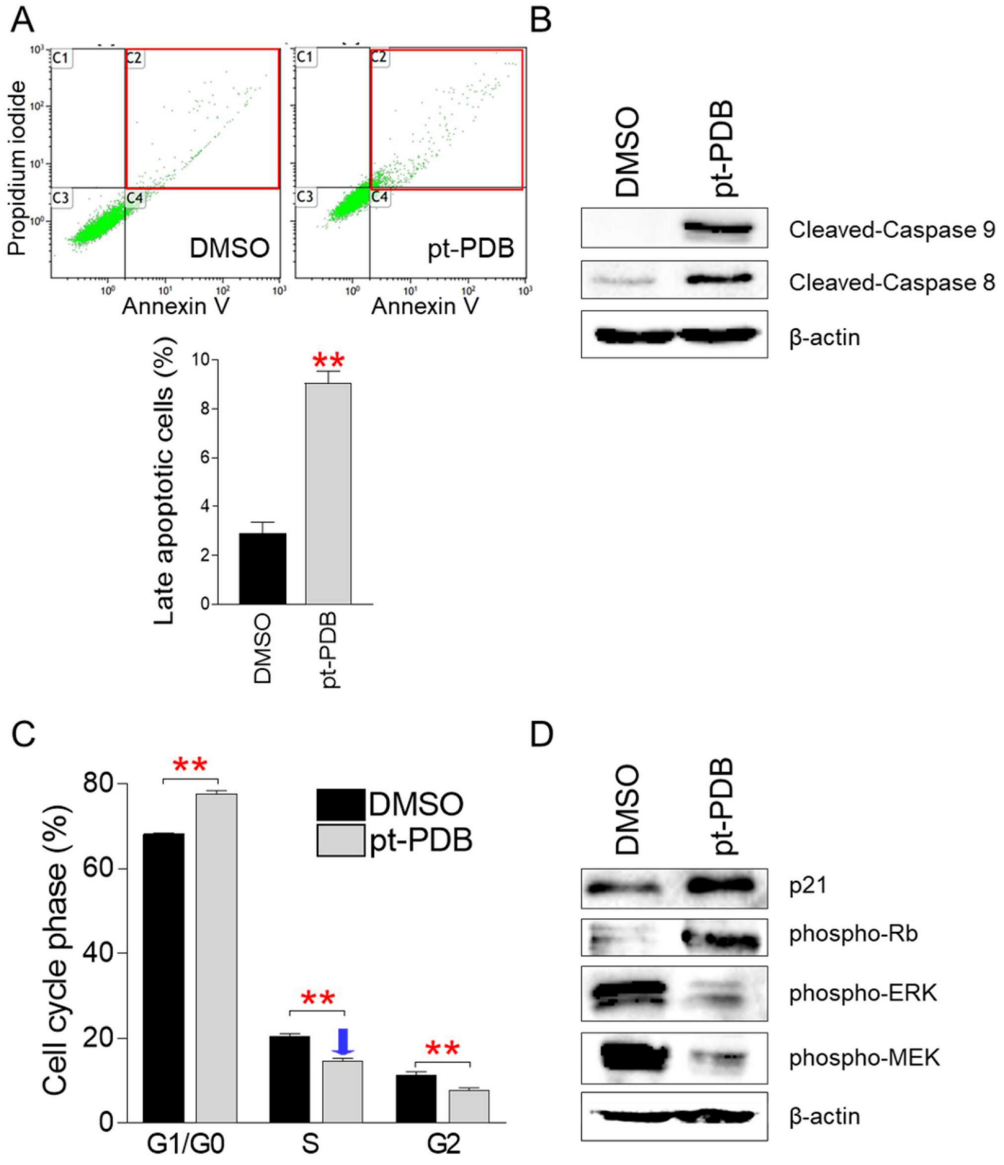
pt-PDB causes apoptosis and S-phase arrest in order to inhibit cell proliferation. (A) Using annexin V and propidium iodide labeling, flow cytometric examination of apoptotic cells was carried out. Late apoptotic cell populations are represented by red squares. **P < 0.01, student's t-test. Means ± S.D., *n* = 3. (B) Effect of pt-PDB on the level of protein expression in the apoptotic pathway. Caspase 9 and Caspase 8 in their cleaved form. (C) Using PI labeling, flow cytometric examination of cell cycle was carried out. **P < 0.01, student's t-test. Means ± S.D., *n* = 3. (D) Effect of pt-PDB on the level of protein expression in the cell cycle pathway. p21Cip1 (p21), phospho-Rb, phospho-ERK, and phospho-MEK.

Observation of dead cells by pt-PDB treatment led us to investigate the proportion of cells killed by apoptosis among dead cells, because apoptosis is a programmed cell death process, and effective elimination of cancer cells by apoptosis has been a critical goal of cancer treatment 33. Apoptotic cells were discriminated from necrotic cells using annexin V/propidium iodide (PI) staining 34. Compared to the DMSO control, pt-PDB treatment significantly increased the proportion of late apoptotic cells that were annexin V/PI-double positive (Fig. 3A). These results suggest that pt-PDB induces the release of pro-inflammatory cellular contents and membrane damage leading to late apoptotic cells.

To further support the induction of apoptosis by pt-PDB, we examined the cleavage of caspase 9, which acts as an initiator of intrinsic apoptosis, and caspase 8, which acts as an initiator of death receptor-induced apoptosis 35. Caspase 9 cleavage was much higher in pt-PDB compared to the DMSO control, indicating that the pt-PDB initiated intrinsic apoptosis (Fig. 3B). In addition, cleavage of caspase 8 was much higher in pt-PDB compared to DMSO group, indicating that pt-PDB executed death receptor-induced apoptosis (Fig. 3B).

Due to aberrant activation of cell cycle components, cancers exhibit deregulated cell proliferation 25. Therefore, one of the key indicators for demonstrating the efficacy of anticancer effect is to determine whether a candidate anticancer agent induces cell cycle arrest, particularly S-phase arrest 26, 27. We then investigated whether pt-PDB inhibits cell proliferation through triggering S-phase arrest. Compared to the DMSO control, cells treated with pt-PDB significantly increased the percentage of cells in G1/G0 phase from 68.1% to 77.6% (Fig. 3C). Moreover, pt-PDB significantly lowered the S-phase percentage from 20.5% to 14.6%, suggesting that pt-PDB inhibits cell proliferation by causing S-phase arrest (Fig. 3C).

The confirmation that pt-PDB inhibits cancer cell proliferation by inducing S-phase arrest led us to investigate how pt-PDB affects the expression levels of cell cycle regulatory proteins. p21Cip1 (p21) and phosphorylated form of retinoblastoma (p-Rb) are generally known proteins involved in S-phase arrest 36, 37. Therefore, we examined the effect of pt-PDB treatment in the expression levels of p21 and p-Rb. Both p21 and p-Rb expressions were markedly increased by pt-PDB treatment, indicating that pt-PDB triggers S-phase arrest through activating p21-RB pathways (Fig. 3D). Extracellular signal-regulated kinase (ERK) and mitogen-activated protein kinase (MEK) are important controllers of S-phase entry signals during the cell cycle 38, 39. Therefore, we investigated the expression levels of phospho-ERK (p-ERK), an active version of ERK, and phospho-MEK (p-MEK), an activated form of MEK. After pt-PDB treatment, the expression of p-ERK and p-MEK decreased, indicating pt-PDB treatment prevented the cell cycle progression (Fig. 3D).

### pt-PDB prevents the proliferation of cancer cells derived from lung cancer

**Figure 4:**
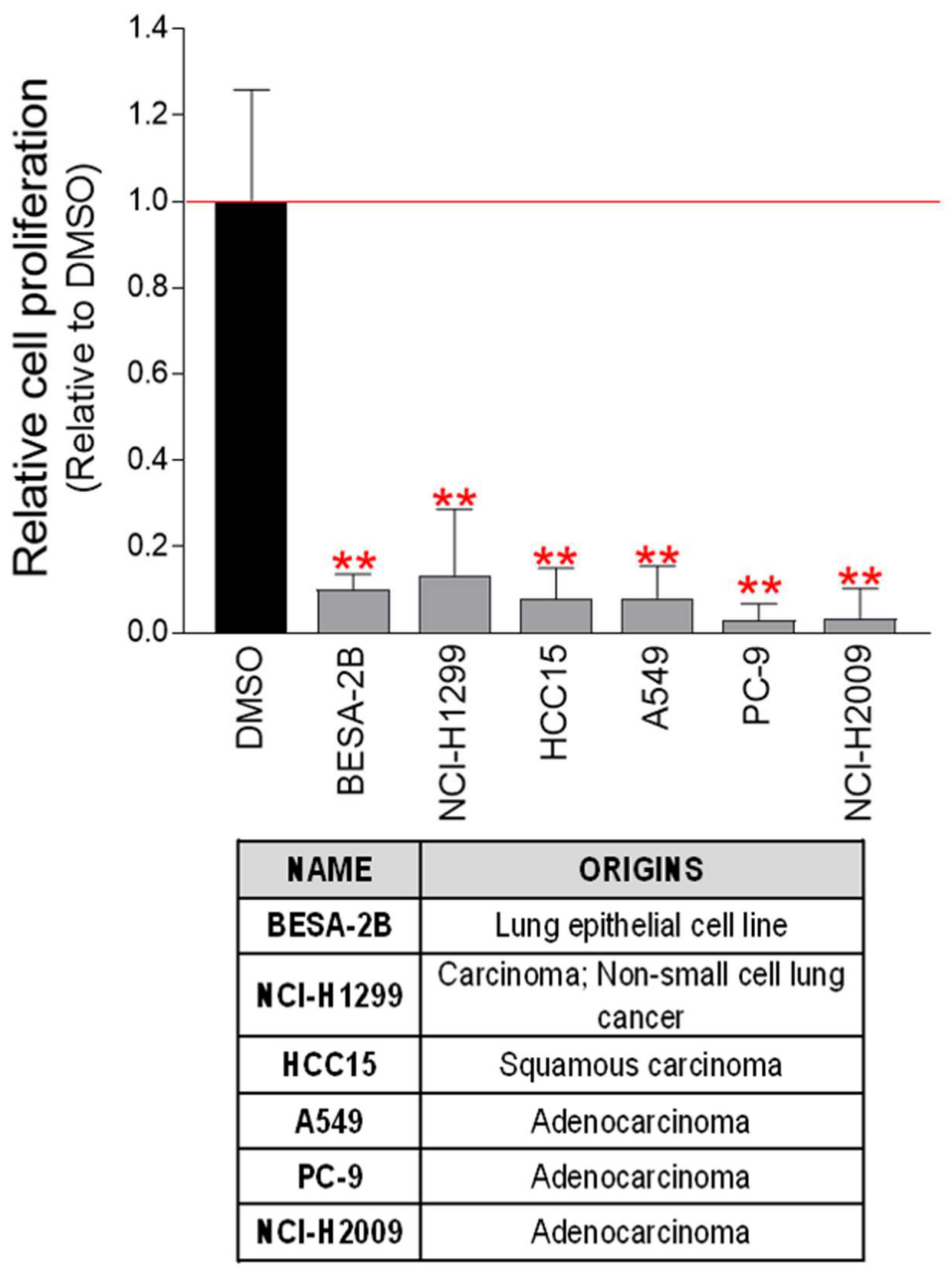
pt-PDB has an anticancer effect on several lung cancer-derived cells. Six lung cancer-derived cells were exposed to pt-PDB diluted to a concentration of 0.1 mg/ml in the medium. The cell proliferation inhibitory effect was assessed on the 4th day of treatment. **P < 0.01, student t-test. Means ± S.D., *n* = 10.

Validation of the anticancer effect of pt-PDB in cervical cancer-derived Hela cells led us to investigate whether the anticancer activity of pt-PDB extends to other cancer cell lines derived from surgically resected human tumor samples. Therefore, the effect of pt-PDB was investigated in six cancer cell lines derived from human lung cancer (BESA-2B, NCI-H1299, HCC15, A549, PC-9, NCI-H2009). Cancer cell growth was significantly reduced in all six lung cancer cell lines treated with pt-PDB (Fig. 4). These results suggest that the anticancer properties of pt-PDB can be used not only in cells derived from a single cancer species, but also in cell lines derived from other types of cancer.

### pt-PDB does not increase the proportion of SP cells

**Figure 5:**
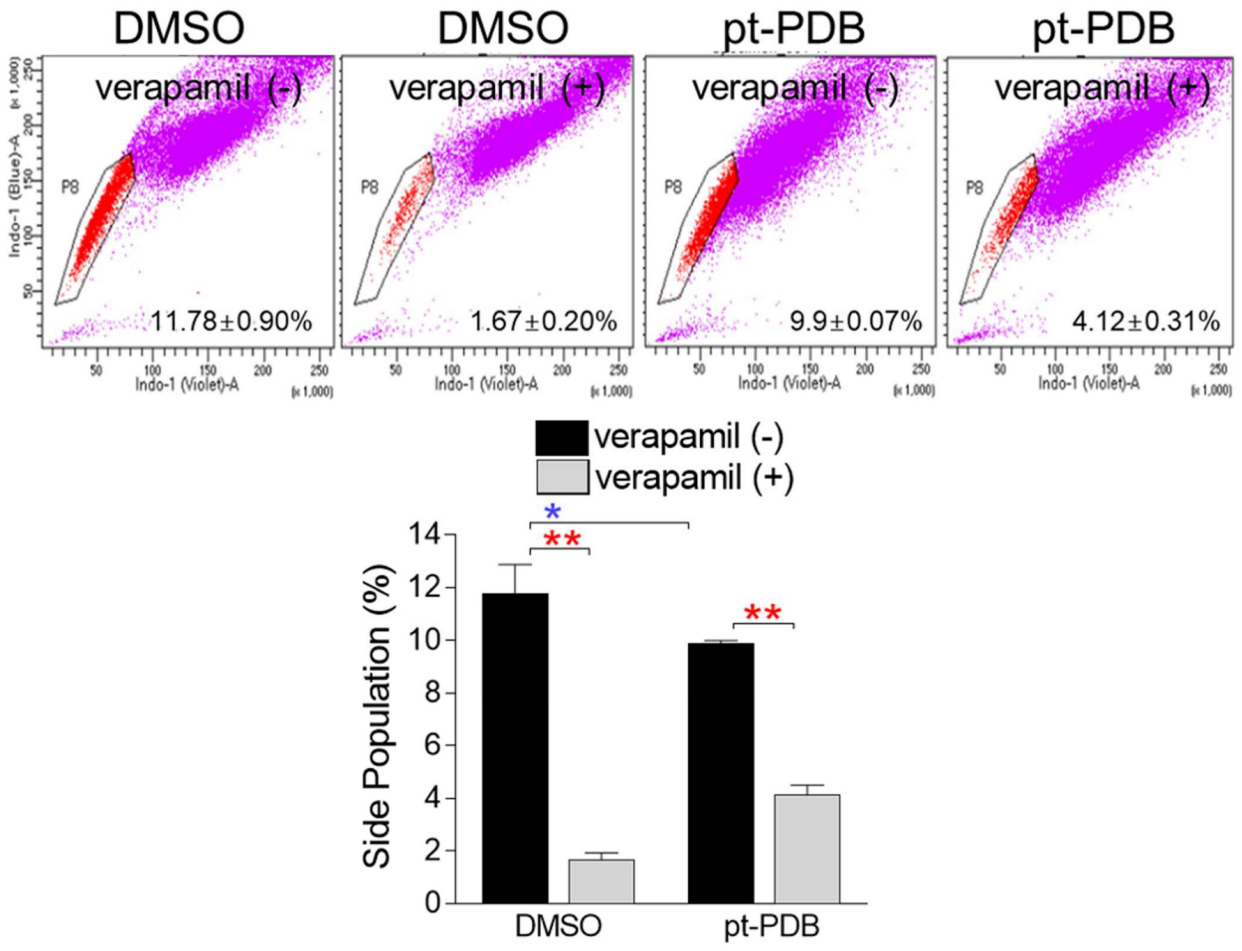
pt-PDB does not increase the proportion of side population (SP) cells. Cells were stained using Hoechst 33342. Verapamil was used to prevent the efflux of Hoechst 33342. The side population was analyzed using flow cytometry. Side populations in the flow chart were placed using pentagon gates. *P < 0.05, **P < 0.01, student t-test. Means ± S.D., *n* = 3.

The emergence of malignant tumors resistant to chemotherapy is one of the major problems that make cancer treatment difficult 11. SP cells, like chemoresistant cells, have the property of effluxing drugs via ABC transporters 40. When stained with Hoechst 33342 dye, SP cells are distinguished from other cells because they rapidly efflux dye via ABC transporters 41. However, SP cells lose this ability when exposed to ABC transporter inhibitors such as verapamil as they are unable to efflux the dye through the transporter 41. Paclitaxel, the most popular anticancer drug, is very effective in treating various cancers, but like other chemotherapeutic agents, it also has a fatal disadvantage of inducing drug resistance by increasing the proportion of SP cells 39, 42. We then looked at whether pt-PDB had side effects such as increasing the proportion of SP cell population. SP cells accounted for 11.78% of the total cell number in DMSO control group (Fig. 5). Verapamil significantly reduced the percentage of SP cells in DMSO control, indicating that the selected cells displayed SP cell characteristics (Fig. 5; red asterisk). Moreover, SP cells accounted for 9.9% of the total cell number in pt-PDB treated-group (Fig. 5). Verapamil significantly dropped the percentage of SP cells to 4.11% in pt-PDB group, proving that the selected cells had SP cell properties (Fig. 5; red asterisk). However, when comparing the ratio of SP cells between DMSO and pt-PDB groups, the percentage of SP cells in pt-PDB group was rather decreased compared to DMSO group (Fig. 5; blue asterisk). Taken together, our findings suggest that pt-PDB may be effective in treating chemoresistant cells because it exerts anticancer effects without increasing the proportion of SP cells.

## Discussion

Mushrooms produce secondary metabolites, which offer potential therapeutic benefits, including antioxidant and anticancer abilities 43. Some secondary metabolites have been found to be relatively non-toxic and show potential for use as therapeutic drugs 44. In this study, the mycelia of *P. tuberaster* were cultured using 4 distinct media (DYB, MEB, MYB, PDB). The culture extracts from DYB did not exhibit any anticancer activity, while the extracts from MEB, MYB, and PDB showed significant anticancer activities. This observation aligns with previous studies indicating that different supplements have been found to have a significant impact on mushroom yield and quality including secondary metabolites 45. Notably, the culture extract of PDB showed the highest anticancer activity among all media tested. This can be attributed to the finding that the PDB medium contains only potato starch, whereas the DYB, MEB and MYB medium do not contain potato starch. Based on these results, it can be suggested that a medium containing potato starch creates an optimal medium condition for *P. tuberaster* to produce secondary metabolites with anticancer activity. This study is, to the best of our knowledge, the first to determine optimal media conditions for the formation of secondary metabolites with anticancer activity in *P. tuberaster*. If the composition of the PDB medium is further improved through processes such as spent medium analysis, it will a turning point in establishing medium conditions in which *P. tuberaster* or other mushrooms can generate secondary metabolites having anticancer activity.

Chemotherapy, which eliminates rapidly dividing cancer cells, is one of the most successful treatments for major cancers 46. However, chemotherapy is successful in the early stages of cancer but loses effectiveness when cancer cells become chemoresistant 47. For example, paclitaxel effectively kills cancer cells in the early stages of treatment, but loses its efficacy in the later stages 48. Therefore, the discovery of drugs that do not cause drug resistance will open a new era in cancer treatment. Recent studies have demonstrated that SP cells are sources of drug resistance during chemotherapy 49, 50. SP cells activate drug efflux channels such as ABC transporter to efflux various anticancer drugs. As a result, SP cells develop resistance to chemotherapy and eventually contribute to recurrent cancers 51, 52. The novelty of this study lies in the fact that pt-PDB has anticancer properties, but unlike conventional anticancer drugs, it does not increase the proportion of SP cells. More research is needed to determine which active ingredient present in pt-PDB plays a key role in producing these effects, but the active ingredient alone or in combination with other anticancer drugs will be very effective in treating chemoresistant cancers.

Eukaryotic cells can kill themselves through the process of apoptosis without triggering an inflammatory response 53. Since avoidance of apoptosis is an important characteristic of cancer cells, induction of apoptosis has been used as an effective method to prevent cell proliferation 33. Another hallmark of cancer cells is dysregulation of cell cycle progression due to defects in regulatory proteins that drive cell cycle progression 54. Therefore, controlling cell cycle progression has been considered a crucial objective of cancer treatment 54. Here, pt-PDB induced apoptosis and S-phase arrest to kill cancer cells. These favorable results were not limited to a specific type of cancer cell and were effective for other cancer cell types as well. Based on the results that pt-PDB effectively controls the two most prominent characteristics of cancer cells, we suggest that pt-PDB should be first considered as an alternative treatment option for cancer treatment.

A new method called mycelial liquid culture allows mycelia to grow in liquid without the need for traditional agar plates 55, 56. Compared to commonly used solid medium culture, mycelial liquid culture has the advantage of requiring less manufacturing time and culture space 56. Mycelial liquid culture also allows rapid colonization of mycelia and scale-up of cell cultures, leading to low-cost mass production of secondary metabolites 57. Therefore, the production of high value-added materials such as polysaccharides, pharmaceuticals, and enzymes is achieved through liquid culture of mycelia 56. Here, liquid culture of *P. tuberaster* in PDB medium produced secondary metabolites with anticancer potential. However, since single liquid cultures were performed on a laboratory scale, the total amount of secondary metabolites obtained was limited. If a large-scale liquid culture production method is established by changing process parameters such as pH, temperature, dissolved oxygen, and agitation speed, the amount of secondary metabolites produced during single liquid cultures can be raised to a level suitable for commercial production.

In summary, *P. tuberaster* mycelium culture extracts effectively inhibited cancer cell growth by inducing apoptosis and S-phase arrest. The significance of its anticancer effect was reinforced by the finding that pt-PDB did not increase the proportion of SP cells. These novel anticancer candidates can be produced at low cost through mass culture and can be used clinically for the treatment of cancer, especially chemoresistant malignancies.

## Figures and Tables

**Table 1:** Names and components of the four media used in this study

Media name	Components
PDB	• dextrose (215530; BD Difco, Franklin, NJ, USA) 20 g/l • potato starch (S2004; Sigma, Saint Louis, MO, USA) 4 g/l
MYB	• malt extract (218630; BD Difco) 20 g/l • yeast extract (212750; BD Difco) 2g/l
MEB	• maltose (216830; BD Difco) 1.8 g/l • dextrose (215530; BD Difco) 6 g/l • malt extract (218630; BD Difco) 6 g/l • yeast extract (212750; BD Difco) 2g/l
DYB	• dextrose (215530; BD Difco) 20 g/l • yeast extract (212750; BD Difco) 2g/l

**Table 2:** Names and details of the 7 cell lines used in this study

Cell line name	Catalogue number	Source
HeLa cells	A1100001; Thermo Fisher Scientific, Waltham, MA, USA	Cells were established from the tumor of a 31-year-old woman with a cervical carcinoma 20.
BEAS-2B	CRL-9609™; American Type Culture Collection, Manassas, VA, USA	Cells were established from normal bronchial epithelium obtained from autopsy of non-cancerous individuals. Then, cells were infected with a replication-defective SV40/adenovirus 12 hybrid and cloned 21.
NCI-H1299	CRL-5803™; American Type Culture Collection	Cells were established from the tumor of a 43-year-old man with carcinoma 22.
HCC15	ACC 496; Deutsche Sammlung von Mikroorganismen und Zellkulturen, Braunschweig, German	Cells were established from the tumor of a 47-year-old man with non-small cell lung carcinoma (subtype squamous carcinoma) 23.
A549	CRL-185™; American Type Culture Collection	Cells were established from the lung tissue of a 58-year-old man with lung cancer 24.
PC-9	90071810; European Collection of Authenticated Cell Cultures, Salisbury, UK	Cells were established from the tumor of a 45-year-old man with lung adenocarcinoma 25.
NCI-H2009	CRL-5911™; American Type Culture Collection	Cells were established from the tumor of a 68-year-old woman with stage 4 adenocarcinoma 26.
